# Lacto-Phosphate of Lime

**Published:** 1874-11

**Authors:** 


### Editorial.

### LACTO-PHOSPHATE OF LIME.

Some experiments have been made during the past ten or twelve months, with lacto-phosphate of lime, and lactic acid and Phosphate of Lime, for the treatment of exposed and diseased pulps of teeth; and so satisfactory have been the results, that some further notice of it seems now to be called for, and many enquiries are made as to the cases in which it is applicable, and the method of using it.

We have been using it since March last and have been much pleased with its operation in most cases in which there was any hope at all.

In attempting to give a description of its use and results we will quote from a letter from Dr. J. E. Cravens, of Kansas City, Mo., to Dr. Rehwinkle, of Ohio. So far as we know, Dr. Cravens was the first dentist to suggest and use it. It will be observed that he speaks of lacto-phosphate, while our experiments have been chiefly with lactic acid and phosphate of lime; but to his experience. He says:

"I believe I mentioned to you in a previous letter, that I had an interesting case on hand. A young man of say twenty years, was in Denver, Col., Sept., 1873, and was attacked with a violent tooth-ache. The pain increased so that it made him sick, and his father, being there at the time, would not have the tooth removed; but brought his son home to this city at once. On arriving here, the young man came at once to consult me. I found the troublesome tooth to be the left superior first molar, with an anterior proximal cavity. In this cavity I found a vast quantity of white flocks or bundles of fibrous debris which I partially removed. In doing so I accidentally uncovered the pulp at two points. I applied tincture opia to relieve the pain, bled gum and pulp freely. I then applied paste of lacto-phosphate of lime, with the paste and protecting cement in situ. I never used an atom of any of the usual agents for pulp exposure in this case. The first sitting I applied laudanum simply as an anodyne, not using it afterward. I renewed the application of the paste several times, and on two other occasions it caused some pain, usually beginning an hour or more after the application was made. I found on examination, that the pain only occurred at those times when I failed to use the rubber dam, and further investigation proved that the os-artificiel insulator had got diluted with mucous and general moisture from gum at cervical margin of the cavity; when the os-artificiel yet soft in consequence of which the saliva soon worked its way into the lactic acid and made a *row*.

The second sitting of this case disclosed a protrusion of the pulp through each exposure and both protrusions badly strangulated. I excised both strangulated protrusions, and then removed the paste. At the first I changed the paste every week. Then I changed no oftener than once in two or three weeks. The soft fibrous mass that I had allowed to remain in the cavity was so hard by the Holidays that it cut like soft slate. The sensitiveness was nearly gone except right between and close round the former exposures, one of which was then closed up nicely. During the month of January I renewed the application of the paste for the last time prior to our intended visit to St. Louis on the 3d inst. The young man's tooth had no pulp exposure in it at all when that last application was made.

My faith is stronger than ever in lacto-phosphate of lime as a remedy for pulp exposures, sensitive or half decomposed dentine. I am grateful to find your 'abiding faith' for it has given me more courage and hope than anything other than my own experiments and experience."

With the lactic acid and phosphate our results have been in the same direction as that above described. In the majority of cases when applied to an aching pulp it has soon ceased to be painful and so remained during and after subsequent applications. In a few instances it has provoked irritation and severe and protracted pain, but it was on those cases where the periosteum was affected in connection with diseased pulps.

The following is an illustration of many cases in our experience:

Mr. B. presented the first right superior molar much decayed on the posterior proximal, involving the posterior lingual surface. The tooth had been painful for several days. Removed the debris and decay, when a large cavity was shown with rather a large pulp exposure. It was washed out, dried and a paste of lactic acid and phosphate of lime, of proper consistence, was applied; and the cavity filled with a hard adhesive wax. The patient at once remarked that the tooth felt better than for days past; in fact, there was no pain or uneasiness. At the end of three days it was removed, cleaned out, examined and presented a good appearance. A second application was made which remained a day or two with the same result; then this was removed and for a test, carbolic acid was applied; pain immediately occurred and continued, though not very severe, for twenty-four hours; when it was removed and the lactic acid and phosphate of lime applied with at once an entire cessation of pain, the patient remarking, "Now, that feels allright." This remained several days, it was then removed and another application of the same made, and the cavity filled with Guillois' cement, and from that time to this, about five months, the tooth has been as free from any unfavorable symptoms as though it had never been decayed or diseased. The pulp is evidently living.

This is a fair example of many cases. So encouraging has this treatment been in our hands that we have no hesitancy in advising all to try it.

The lacto-phosphate as it has been prepared, readily decomposes, when it is unfit for use, so we prefer to keep the lactic acid and phosphate of lime separate, and mix them at the time of using.

We shall be greatly gratified on several accounts if this proves to be a valuable and efficient agent in the treatment of exposed pulps with a view to their preservation and restoration to normal function.

